# Evolutionary and structural annotation of disease-associated mutations in human aminoacyl-tRNA synthetases

**DOI:** 10.1186/1471-2164-15-1063

**Published:** 2014-12-04

**Authors:** Manish Datt, Amit Sharma

**Affiliations:** Structural and Computational Biology group, International Center for Genetic Engineering and Biotechnology, New Delhi, 110067 India

**Keywords:** Aminoacyl-tRNA synthetases, Mutations, Human diseases

## Abstract

**Background:**

Mutation(s) in proteins are a natural byproduct of evolution but can also cause serious diseases. Aminoacyl-tRNA synthetases (aaRSs) are indispensable components of all cellular protein translational machineries, and in humans they drive translation in both cytoplasm and mitochondria. Mutations in aaRSs have been implicated in a plethora of diseases including neurological conditions, metabolic disorders and cancer.

**Results:**

We have developed an algorithmic approach for genome-wide analyses of sequence substitutions that combines evolutionary, structural and functional information. This pipeline enabled us to super-annotate human aaRS mutations and analyze their linkage to health disorders. Our data suggest that in some but not all cases, aaRS mutations occur in functional and structural sectors where they can manifest their pathological effects by altering enzyme activity or causing structural instability. Further, mutations appear in both solvent exposed and buried regions of aaRSs indicating that these alterations could lead to dysfunctional enzymes resulting in abnormal protein translation routines by affecting inter-molecular interactions or by disruption of non-bonded interactions. Overall, the prevalence of mutations is much higher in mitochondrial aaRSs, and the two most often mutated aaRSs are mitochondrial glutamyl-tRNA synthetase and dual localized glycyl-tRNA synthetase. Out of 63 mutations annotated in this work, only 12 (~20%) were observed in regions that could directly affect aminoacylation activity via either binding to ATP/amino-acid, tRNA or by involvement in dimerization. Mutations in structural cores or at potential biomolecular interfaces account for ~55% mutations while remaining mutations (~25%) remain structurally un-annotated.

**Conclusion:**

This work provides a comprehensive structural framework within which most defective human aaRSs have been structurally analyzed. The methodology described here could be employed to annotate mutations in other protein families in a high-throughput manner.

**Electronic supplementary material:**

The online version of this article (doi:10.1186/1471-2164-15-1063) contains supplementary material, which is available to authorized users.

## Background

Mutation(s) in housekeeping proteins often lead to serious ailments in humans [1]. Analysis of molecular bases of mutations that lead to dysfunctional proteins is an important step towards acquiring a detailed understanding of genetic disorders. Several studies have annotated disease-causing mutations in the human genome [2, 3]. The knowledgebase developed through these will be useful in guiding and orienting translational therapeutic research [4]. Aminoacyl-tRNA synthetases (aaRSs) drive cellular protein translation by catalyzing ligation of cognate tRNA with amino-acid for use in ribosomal protein synthesis [5]. The catalytic reaction follows a two step process as follows:

AA + ATP → AMP-AA + PP_i_

AMP-AA + tRNA → tRNA^AA^ + AMP

In the first step, amino acid (AA) is charged with ATP and pyrophosphate (PP_i_) is released. The second step involves charging of cognate tRNA with amino acid and release of AMP. Evolution of a dedicated editing domain in some aaRSs highlights the stringent requirement for fidelity of these reactions [6, 7]. In addition to these translational functions, aaRSs also participates in many other important physiological activities such as translational and transcriptional regulation, signal transduction, cell migration, angiogenesis, inflammation, and tumourigenesis [8–10]. Indeed, in pathogenic systems, the numerous attributes of aaRSs are just being uncovered [11, 12]. Hence, aaRSs with their exquisite range of canonical and non-canonical functions constitute an important subset of proteomes. These enzymes have a modular architecture with separate domains for catalysis, tRNA binding and editing [13]. Based on the domain architecture and tRNA binding modes, aaRSs have been classified into two groups – Class I and II [14]. Class I aaRSs in humans are monomeric except for YRS and WRS that are dimeric. Class II aaRSs in humans are dimeric enzymes except for ARS which is monomeric. A penta-motif (‘KMSKS’) and a tetra-motif (‘HIGH’) are the two evolutionarily conserved motifs that mediate ATP binding in Class I enzymes [15–17]. Class II aaRSs have three conserved motifs – motif1, motif2, and motif3 – which facilitate ATP and amino-acid binding to the active site of the enzyme [18].

In humans, except for GRS and KRS, separate set of genes within the nuclear genome encode for cytoplasmic and mitochondrial aaRSs [19]. Cytoplasmic and mitochondrial GRSs are generated from distinct translation initiation sites on the same gene (GARS) [20] while for KRS alternate spliced products of same gene (KARS) undergo differential sub-cellular localization [21]. The human genome lacks gene for mitochondrial QRS and it has been hypothesized that mitochondrial Gln-tRNA^Gln^ is synthesized in two steps – first, tRNA^Gln^ is misacylated to Glu-tRNA^Gln^ and second, generation of Gln-tRNA^Gln^ by the action of glutaminyl amidotransferases [22]. Thus, the human genome in total has 37 genes coding for aaRSs. Human mitochondrial aaRS are encoded by nuclear genome and are trafficked to the organelle. Ten mitochondrial and four cytoplasmic aaRSs have been implicated in human diseases so far [23]. The Protein Data Bank (PDB) has structural representatives for all 20 members of aaRS family. For 11 aaRSs, crystallographic structures of human proteins are also available. Further, crystal structures have been solved to elucidate mechanism of interaction of these proteins with other biomolecules like ATP [24], tRNA [25] and other proteins [26]. Owing to the multitude of functions that aaRSs perform, it is no surprise that mutations in these proteins often prove to be deleterious and lead to diseases [27, 28].

Over the years, many mutations have been identified in different aaRSs [28]. These mutations results in dysfunctional aaRSs leading to various neurological and metabolic disorder such as Charcot-Marie-Tooth (CMT) disease, Amyotrophic Lateral Sclerosis, cancer, and diabetes [9, 28]. The repertoire of sequence and structural information for aaRSs provides an opportunity to investigate structural distribution and functional relevance for mutations in these proteins leading to diseases in humans. In this study we have systematically analyzed all aaRS mutations in cytoplasmic and mitochondrial enzyme copies. We have evaluated disease-associated mutations within aaRSs in context of their structural features and sequence conservation. Properties such as local secondary structure at the site of mutations along with solvent accessibility profiles have been investigated to evaluate potential perturbations caused by mutations. In addition, evolutionary sequence conservation information has been used to annotate all mutant sites. The possibility of mutations to influence intra- and inter- molecular interactions mediated by aaRSs has also been examined. Our methodology can be effectively used to predict whether a particular mutation in an aaRS could directly affect aminoacylation activity or alter some other attribute such as interaction with biomolecules. The results presented advance our understanding of mutation driven pathologies in humans. Further, this study offers a mutation annotation pipeline which is available for academic groups in the form of python scripts. We believe that the methodology outlined here would prove useful for examining mutations within different protein families in a high-throughput manner wherever sequence and structural information is available.

## Methods

Throughout the manuscript aaRSs are referred to as ‘XRS’ where X is the single letter code for corresponding amino acid e.g. alanyl-tRNA synthetase is mentioned as ARS. The gene name for an aaRS is referred to as ‘*XARS*’ where *X* is the single letter code for corresponding amino acid e.g. the gene for alanyl-tRNA synthetase is mentioned as *AARS*. The gene name for mitochondrial proteins has ‘2’ as suffix e.g. gene names for cytoplasmic and mitochondrial ARS are *AARS* and *AARS2* respectively. Mutations in *GARS* and *KARS* genes are discussed under the cytoplasmic aaRSs although these two genes encode for both cytoplasmic and mitochondrial copies of GRS and KRS respectively. The aaRS mutations annotated in this manuscript were retrieved from the literature published till first quarter of 2014. The mutational annotation pipeline ran as follows (Additional file 1: Figure S1): these analyses required sequences of human aaRSs and a list of substitution mutations in each enzyme. Structural homologues for human aaRSs were identified using BLAST searches against PDB. These structures were then used to identify residues participating in inter-molecular interactions such as binding with ATP, amino acid, tRNA and oligomer formation. In addition, secondary structure and solvent accessibility for residues in structural homologues corresponding to mutated residue were calculated. Finally, an annotated pairwise sequence alignment between human aaRS and structural homologue was constructed. Evolutionary conservation at mutational sites was also calculated. In general, we have annotated aaRSs mutations into four categories: (a) those likely to abrogate or disturb ligand or tRNA binding due to direct contacts with substrates/products, (b) those that are part of aaRS structural core and where a change may directly affect enzyme folding/stability, (c) those that occur at protein surfaces which will end up being interfaces during assembly of oligomers, and (d) those that do not fall into any of the above. In case of latter, experimentation and validation is required to understand the mechanistic basis of mutational effects.

### Calculation of intermolecular contacts

Inter-molecular contacts between aaRS and their binding partners (e.g. ATP, amino-acid, tRNA, homo-dimeric partner) were calculated using distance-based approaches. Any aaRS residue within 4 Å of the binding partner was considered to be participating in intermolecular contacts. These interacting residues were then used to annotate mutations sites.

### Multiple Sequence Alignment (MSA)

#### MSA for each aaRS

Mammalian homologues for each human aaRS were identified in the non-redundant (NR) database using BLAST. Top 100 BLAST hits were selected to generate MSA specific for each aaRS. These alignments were used to evaluate the phylogenetic distribution of residues at mutation location.

#### MSA for each class of aaRS

Non-redundant datasets of class I and II aaRS crystal structures were prepared with total of 57 and 39 structures respectively. These were prepared with sequence similarity cutoff of ~90% using tools available at Protein Data Bank. Using this dataset, structure-based multiple sequence alignment was generated for each enzyme class separately. Structural alignment was calculated using TM-align program [29] as implemented in T-Coffee package [30]. Both class I and II aaRS, independently, have very similar folds within their classes – this allowed generation of a structure-guided sequence profile for each. These structure-based MSAs were used to calculate residue conservation score at different positions in the sequence using blosum62 matrix. Positional conservation in structure-based MSA was divided into four groups – <40%, 40%-60%, 60%-80%, and >80%. Alignment positions with scores that could fall in two intervals were assigned to an interval with identical lower limit e.g. a score of 60% would be classified under 60-80% interval.

### Pair-wise sequence alignment

Human aaRS sequences were aligned with sequences of 3D structures using T-Coffee [30]. Sites of mutations were highlighted in the query sequences using in-house python scripts. The interacting residues in aaRS identified above were highlighted in the sequence data. Residues that emanate from PDB file were color-coded based on conservation scores from the MSA calculated above. Distribution of residues at the site of specific mutation was also calculated from MSA. Human aaRSs lacking crystal structure information were not used for structure-based MSA, and instead the location of corresponding residue from homologous structure in pairwise alignment was used to calculate structural conservation.

### Calculations of structural features from tertiary structure

#### Secondary structure

Formatted files for human aaRSs were retrieved from PDB, and DSSP program was used to assign secondary structures – which were classified as α-helix, 3_10_ helix, β-strand, turn or unassigned.

#### Solvent accessibility calculations

Residue solvent accessibility (SA) was calculated using DSSP. Relative solvent accessibility (RSA) for a residue in protein structure is defined as:


Based on RSA values, residues were classified as solvent accessible or buried using following criteria. If for a particular residue the value of RSA was >20% then it was considered solvent accessible else buried. PyMol was used for visual analyses of 3D structures [31].

## Results and discussion

### Mutational landscape of cytoplasmic aaRSs

**Figure 1:**
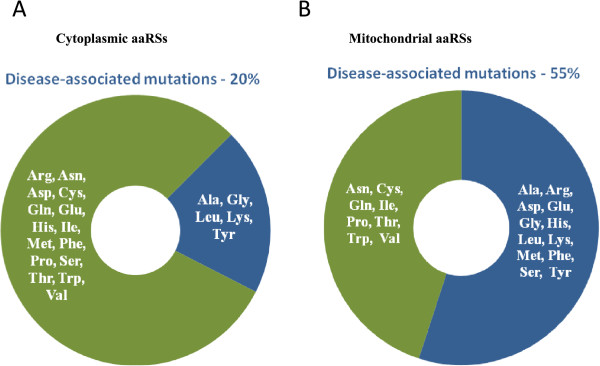
**Distribution of aaRSs within cytoplasm (A) and mitochondria (B) for which mutations have been experimentally identified (blue background) – lack of mutation (green background).** Mutant ARS, LRS and YRS from cytoplasm and mitochondria are associated with diseases. The mitochondrial and cytoplasmic isoforms of GRS and KRS are encoded by the same gene i.e. GARS and KARS respectively.

**Figure 2:**
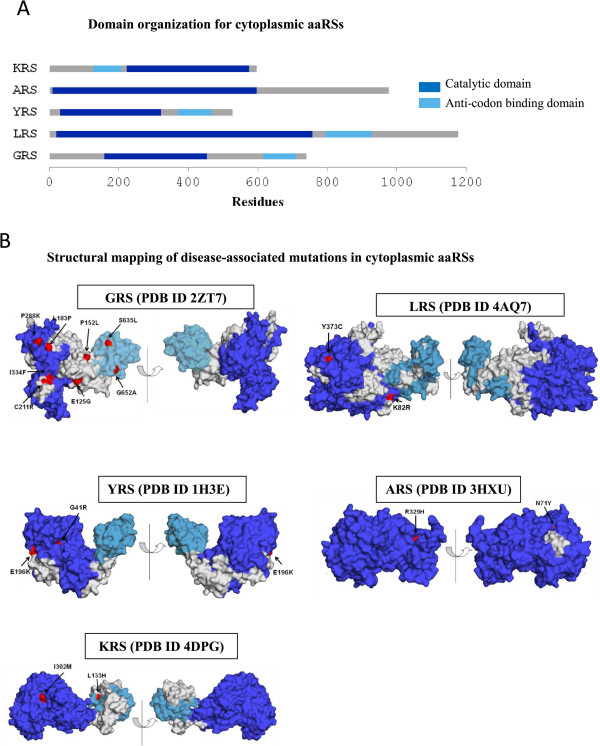
**Disease-associated mutations in human cytoplasmic aaRSs. (A)** Schematic representation of domain architecture of human cytoplasmic aaRSs in which disease-associated mutations have been identified. Catalytic domain and anticodon binding domain are in blue and cyan respectively. Domain boundaries for aaRS sequences were calculated using Pfam server. **(B)** Mutations (red) in human cytoplasmic aaRSs mapped on the crystal structures. Different domains in structures have been color-coded corresponding to **(A)**.

Disease-associated mutations have been identified in four cytoplasmic aaRS enzymes so far [28]. Mutations in LRS/YRS within class I and ARS/GRS/KRS within class II lead to various human diseases (Table 1 and Figure 1A). Each point mutation in a given cytoplasmic aaRS has been independently associated with disease. Figure 2A shows schematic representation of domain architectures for these. Briefly:

**Table 1:** **List of disease-associated mutations in cytoplasmic aaRSs. In case of GRS and KRS, one gene encodes for cytoplasmic and mitochondrial isoforms**

aaRS (Class)	ID (oligomer)	Substitution mutation(s)	Disease/affected organ
GRS (II)	P41250 (Dimer)	Ala111Val, Glu125Gly, Pro152Leu, Cys211Arg, Leu183Pro, Pro288Lys, Gly294Arg, Ile334Phe, His472Arg, Asp554Asn, Gly580Arg, Ser635Leu, Gly652Ala	Distal hereditary motor neuropathy (dHMN) or distal spinal muscular atrophy (dSMA)/neurons
LRS (I)	Q9P2J5 (Monomer)	Lys82Arg, Tyr373Cys	Infantile hepatopathy/liver in new born babies
YRS (I)	P54577 (Dimer)	Gly41Arg, Glu196Lys	Dominant intermediate Charcot-Marie-Tooth disorder type C (DI-CMTC)/neurons
ARS (II)	P49588 (Monomer)	Asn71Tyr, Arg329His, Glu778Ala, Asp893Asn	dHMN, CMT type 2 N/neurons
KRS (II)	Q15046 (Dimer)	Leu133His, Ile302Met, Thr623Ser	CMT/neurons

### Mutations in glycyl-tRNA synthetase that causes neurological disorders

**Figure 3:**
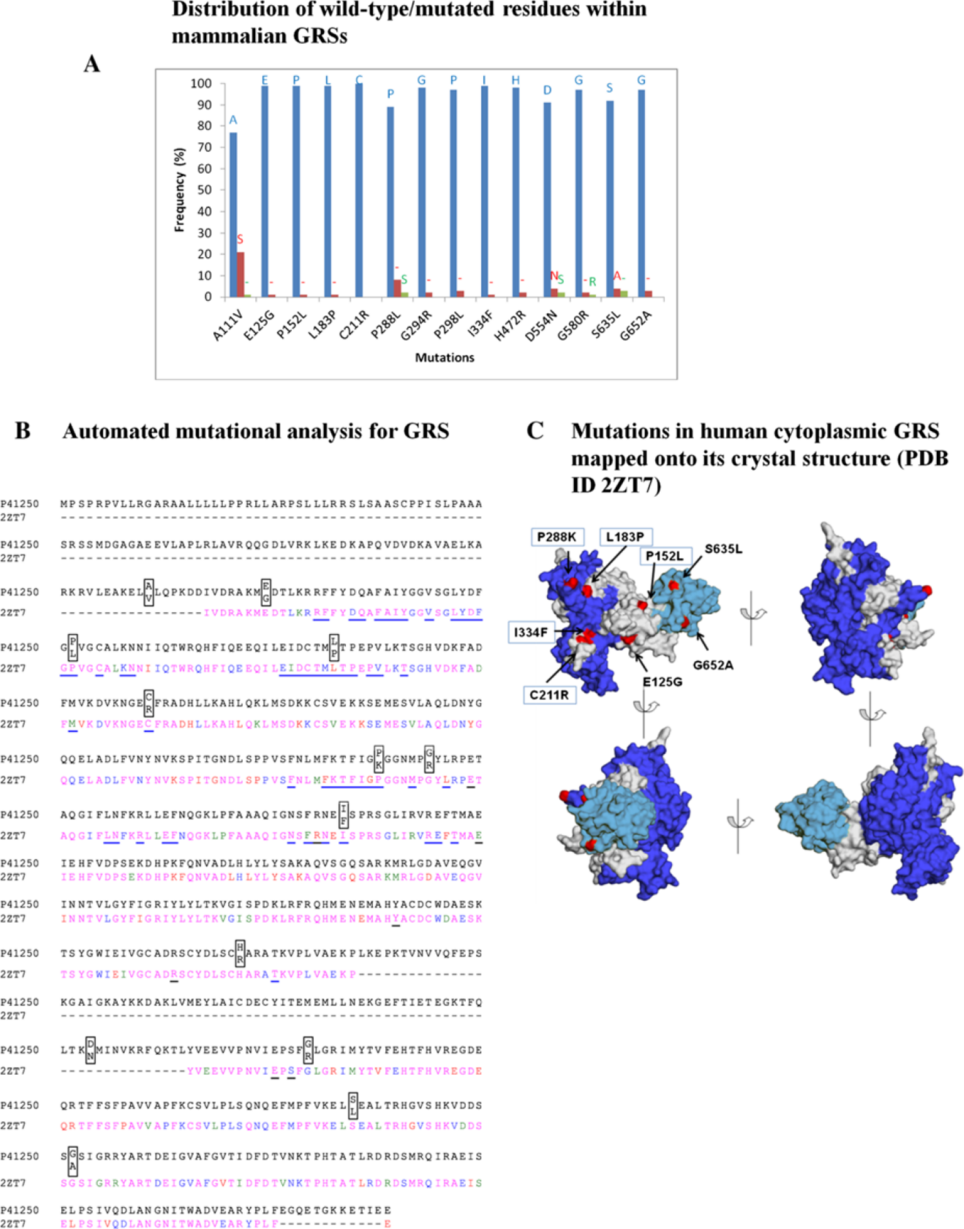
**Annotation for mutations in human cytoplasmic GRS. (A)** Frequency distribution of residues at sites of GRS mutations. The distribution was calculated based on top 100 mammalian homologous sequences identified using BLAST. A ‘-’ represents frequency for gaps in the alignment. **(B)** Alignment between sequence and structure of human cytoplasmic GRS (UniProt ID P41250 and PDB ID 2ZT7) with mutations (boxes). Residues in GRS that interact with ATP/analogue and dimeric interface are underlined in black and blue respectively. Structural conservation for GRS is graded in four categories <40%, 40-60%, 60-80%, and >80% which are highlighted in magenta, blue, green and red respectively. **(C)** GRS mutations and those in dimeric interface are highlighted with a box.

A total of 14 substitution mutations have been identified by exome sequencing of *GARS* gene (Table 1 and Figure 2B). Our phylogenetic and sequence conservation analyses suggest that wild-type residues at mutation sites are highly conserved (Table 2 and Figure 3A). The in-house analysis program output (Figure 3B) displays annotated pairwise sequence alignment between primary sequence and tertiary structure of human GRS where mutations are highlighted in boxes. Juxtaposition of sites in 3D structure of human GRS shows that mutations are distributed throughout GRS (Figure 3C). For Asp554Asn change, the corresponding residue is naturally present in mammalian GRS from *C. cristata* (star-nosed mole). Total of five (of 14) mutations – Pro152Leu, Leu183Pro, Cys211Arg, Pro288Lys and Ile334Phe are directly involved in non-bonded interactions at GRS dimeric interface. Four of these are solvent accessible while Ile334Phe is buried. These observations suggest that Pro152Leu, Leu183Pro, Cys211Arg, Pro288Lys and Ile334Phe substitutions likely affect dimer assembly. Other mutations like Glu125Gly reside in α-helix (H1 in PDB ID 2ZT7) within the catalytic domain of GRS and likely destabilize local secondary structure because of introduction of glycine within α-helical structure. Similarly, the Gly294Arg mutation occurs in a buried location within a β-strand that forms the GRS catalytic domain. Substitution of the smaller glycine residue with a bulky and positively charged arginine may affect GRS folding and stability. Disordered regions within the protein structure have been shown to be critical for mediating interactions with other proteins [32]. The Asp554Asn alteration maps to a disordered region of human GRS but its biochemical effect remains unexplored. Two substitution mutations – Ser635Leu and Gly652Ala – were observed to be part of α-helix and bend structure within the C-terminal domain of GRS where these sites are solvent accessible. It is likely that alterations at these two positions could impair or enhance the ability of GRS to interact with other proteins leading to pathological phenotypes. For the Ala111Val mutation located in GRS N-terminal region, there is no corresponding residue in the crystal structure so its significance remains unexplained. Interestingly, amongst all aaRSs, GRS has the maximum number of disease-associated mutations reported in literature to date. Our data suggest that pathology emanating due to aforementioned GRS mutations may be due to variety of biochemical reasons e.g. alterations/interference in dimer formation and structural instability within GRS core. In summary, GRS mutations fall under three different categories as per our annotation (see Methods): (b), (c), and (d) (Table 3).

**Table 2:** **Annotations for cytoplasmic aaRSs - mutations that were observed to be naturally present in corresponding mammalian aaRSs are highlighted (bold)**

aaRS	Mutation	Sequence spread in homologs (%)	Structure	RSA	Intermolecular interaction	Domain	References
GRS (II)	Ala111Val	‘A’: 77, ‘S’: 21, ‘E’: 1, ‘-’: 1	NA	NA	-	-	[33–39]
	Glu125Gly	‘E’: 84, ‘-’: 10, ‘D’: 6	H	22.7	-	-	
	Pro152Leu	‘P’: 99, ‘-’: 1	H	75.0	Dimerization	-	
	Leu183Pro	‘L’: 90, ‘-’: 8, ‘M’: 1, ‘V’: 1	E	54.3	Dimerization	Catalytic	
	Cys211Arg	‘C’: 100	E	34.8	Dimerization	Catalytic	
	Pro288Lys	‘P’: 98, ‘-’: 2	S	94.1	Dimerization	Catalytic	
	Gly294Arg	‘G’: 81, ‘-’: 16, ‘P’: 1, ‘K’: 1, ‘T’: 1	E	**0.0**	-	Catalytic	
	Pro298Leu	‘P’: 97, ‘-’: 3	S	**0.7**	-	Catalytic	
	Ile334Phe	‘I’: 81, ‘-’: 8, ‘L’: 8, ‘V’: 2, ‘C’: 1	S	**11.2**	Dimerization	Catalytic	
	His472Arg	‘H’: 80, ‘-’: 20	H	**3.3**	-	-	
	Asp554Asn	**‘D’: 81, ‘-’: 8, ‘K’: 7, ‘G’: 2, ‘N’: 1, ‘S’: 1**	-	-	-	-	
	Gly580Arg	‘G’: 92, ‘-’: 8	E	**14.3**	-	-	
	Ser635Leu	‘S’: 83, ‘-’: 8, ‘T’: 7, ‘A’: 2	H	24.6	-	C-terminal	
	Gly652Ala	‘G’: 85, ‘H’: 8, ‘-’: 7	S	46.4	-	C-terminal	
LRS (I)	Lys82Arg	‘K’: 81, ‘-’: 18, ‘N’: 1	-	22.4	-	Catalytic	[40]
	Tyr373Cys	‘Y’: 100	E	21.3	-	Catalytic	
YRS (I)	Gly41Arg	‘G’: 74, ‘-’: 26	E	40.4	ATP/amino-acid binding	Catalytic	[41]
	Glu196Lys	‘E’: 74, ‘-’: 25, ‘P’: 1	H	**16.9**	-	Catalytic	
ARS (I)	Asn71Tyr	‘N’: 97, ‘-’: 3	E	**0.0**	-	Catalytic	[42–44]
	Arg329His	‘R’: 98, ‘-’: 2	H	**4.3**	-	Catalytic	
	Glu778Ala	**‘E’: 79, ‘T’: 10, ‘A’: 6, ‘G’: 2, ‘S’: 2, ‘V’: 1**	-	-	-	-	
	Asp893Asn	**‘D’: 75, ‘P’: 21, ‘N’: 2, ‘X’: 1, ‘-’: 1**	-	-	-	-	
KRS (II)	Leu133His	‘L’: 100	H	**2.4**		Anti-codon binding	[45, 46]
	Ile302Met	‘I’: 100	S	29.5		Catalytic	
	Thr623Ser	‘T’: 77, ‘P’: 16, ‘-’: 4, ‘A’: 1, ‘M’: 1, ‘V’: 1	-	-		-	

RSA values (%) in bold indicate buried positions that were ordered in the crystal structures. Also see Figure 2.

**Table 3:** **Potential defects identified using our mutational annotation pipeline in aaRSs**

Disease	aaRS/mutations	Previous functional annotation for mutation	Structural/functional feature potentially affected (category for mutational annotation)
Charcot-Marie-Tooth disorder (CMT)	GRS (Ala111Val)	No reported annotation	Un-annotated (d)
	GRS (Glu125Gly)	No reported annotation	Dimerization (c)
	GRS (Pro152Leu)	No reported annotation	Dimerization (c)
	GRS (Leu183Pro)	Defective dimerization	Dimerization (c)
	GRS (Cys211Arg)	No reported annotation	Structural stability (b)
	GRS (Pro288Lys)	No reported annotation	Dimerization (c)
	GRS (Gly294Arg)	Defective dimerization	Structural stability (b)
	GRS (Pro298Leu)	No reported annotation	Structural stability (b)
	GRS (Ile334Phe)	No reported annotation	Dimerization (c)
	GRS (His472Arg)	No reported annotation	Structural stability (b)
	GRS (Asp554Asn)	Enhanced dimerization	Un-annotated (d)
	GRS (Gly580Arg)	Enhanced dimerization	Structural stability (b)
	GRS (Ser635Leu)	Enhanced dimerization	Inter-molecular interaction (c)
	GRS (Gly652Ala)	No reported annotation	Inter-molecular interaction (c)
	YRS (Gly41Arg)	Impaired tyrosine activation	ATP/amino-acid binding (a)
	YRS (Glu196Lys)	No reported annotation	Structural stability (b)
	ARS (Asn71Tyr)	Impaired tRNA charging	Structural stability (b)
	ARS (Arg329His)	Imparired tRNA charging	Structural stability (b)
	ARS (Glu778Ala)	No reported annotation	Un-annotated (d)
	ARS (Asp893Asn)	No reported annotation	Un-annotated (d)
	KRS (Leu133His)	Decreased enzyme activity	Structural stability (b)
	KRS (Ile302Met)	No reported annotation	Inter-molecular interactions (c)
	KRS (Thr623Ser)	No reported annotation	Un-annotated (d)
Infantile hepatopathy	LRS (Lys82Arg)	No reported annotation	Un-annotated (d)
	LRS (Tyr373Cys)	No reported annotation	Structural stability (b)
Type-2 diabetes	LRS (His324Gln)	No reported annotation	Structural stability (b)
Perrault syndrome, ovarian failure and hearing loss	LRS (Thr522Asn)	Decreased enzyme activity	Structural stability (b)
	LRS (Thr629Met)	No reported annotation	Structural stability (b)
	HRS (Leu200Val)	Decrease enzyme activity	Structural stability (b)
	HRS (Val368Leu)	Decreased enzyme activity	Structural stability (b)
Myopathy, lactic acidosis, and sideroblastic anemia (MLASA)	YRS (Gly46Asp)	Translational defects	Inter-molecular interactions (c)
	YRS (Phe52Leu)	Abnormal enzyme kinetics	Inter-molecular interactions (c)
HUPRA syndrome	SRS (Asp390Gly)	Decreased enzyme activity	Structural stability (b)
	SRS (Arg402His)	No reported annotation	Structural stability (b)
Infantile cardiomyopathies	ARS (Leu155Arg)	No reported annotation	Un-annotated (d)
	ARS (Arg592Trp)	No reported annotation	Un-annotated (d)
Ponto cerebellar hypoplasia	RRS (Ile9Val)	No reported annotation	Un-annotated (d)
	RRS (Gln12Arg)	No reported annotation	Un-annotated (d)
	RRS (Trp241Arg)	No reported annotation	Structural stability (b)
	RRS (Arg245Gln)	No reported annotation	Structural stability (b)
	RRS (Arg469His)	No reported annotation	tRNA binding (a)
Leukoencephalopathy with brainstem and spinal cord involvement and elevated lactate (LBSL)	ERS (Arg55His)	No reported annotation	Structural stability (b)
	ERS (Glu96Lys)	No reported annotation	Inter-molecular interactions (c)
	ERS (Arg107His)	No reported annotation	Inter-molecular interactions (c)
	ERS (Arg108Trp)	No reported annotation	Inter-molecular interactions (c)
	ERS (Gly110Ser)	No reported annotation	Inter-molecular interactions (c)
	ERS (Lys167Tyr)	No reported annotation	Un-annotated (d)
	ERS (Arg168Gly)	No reported annotation	Un-annotated (d)
	ERS (Gly204Ser)	No reported annotation	Inter-molecular interactions (c)
	ERS (Gly224Ser)	No reported annotation	Inter-molecular interactions (c)
	ERS (Gly317Cys)	No reported annotation	tRNA binding (a)
	ERS (Arg516Gln)	No reported annotation	Structural stability (b)
	DRS (Ser45Gly)	No reported annotation	Dimerization (c)
	DRS (Cys152Phe)	Defective dimerization	Dimerization (c)
	DRS (Arg179His)	No reported annotation	Inter-molecular interactions (c)
	DRS (Leu239Pro)	No reported annotation	Un-annotated (d)
	DRS (Arg263Gln)	Defective dimerization	Dimerization (c)
	DRS (Leu613Phe)	No reported annotation	tRNA binding (a)
	DRS (Leu626Gln)	Decrease enzyme activity	Inter-molecular interactions (c)
	DRS (Leu626Val)	No reported annotation	Inter-molecular interactions (c)
Fatal infantile Alpers encephalopathy, mitochondrial myopathies	FRS (Tyr144Cys)	Defective tRNA binding	Un-annotated (d)
	FRS (Ile329Thr)	Impaired stability/decreased ATP binding	Structural stability (b)
	FRS (Asp391Val)	Impaired stability/decreased Phe binding	Inter-molecular interactions (c)

Mutation are annotated under four categories: (a) those likely to abrogate or disturb ligand or tRNA binding due to direct contacts with substrates/products, (b) those that are part of aaRS structural core and where mutation may directly affect enzyme folding/stability, (c) those that occur at protein surfaces which will end up being interfaces during assembly of oligomers (like dimeric interfaces), and (d) those that do not fall into any of the above and therefore where further experimentation may provide a mechanistic basis for mutational effects.

### Mutations in leucyl-tRNA synthetase that causes infantile hepatopathies

Mutations in the *LARS* gene identified using whole genome sequencing have been associated with life threatening hepatopathies in new born babies [40]. Symptoms include anemia, impaired liver function and overall poor infant development [40]. The two substitution mutations - Lys82Arg and Tyr373Cys – within the LRS catalytic domain have so far been implicated (Table 1 and Figure 2B). We show that the wild-type residues at these two sites are conserved in homologous mammalian LRSs (Table 2). Our analyses suggest a lack of direct participation by residues at these mutation sites in inter-molecular contacts with ATP, amino acid or tRNA. The Tyr373Cys position lies partially buried in a β-strand within the LRS catalytic domain (Figure 2B). In addition, this site has ~80% structural conservation based on non-redundant dataset of 57 Class I aaRSs (Table 2). These results indicate an important contribution of the site 373 in LRS. It is likely that this mutation affects aaRS conformation as substitution of a bulky buried hydrophobic residue (tyrosine) with smaller cysteine in the β-strand could destabilize the local network of non-bonded interactions within the protein structure. The second mutation location of Lys82Arg occurs within the catalytic domain in a buried environment but without a clear hint of its possible structural effects on LRS (Figure 2B). Based on our structural analysis Lys82Arg and Tyr373Cys mutations falls under categories (d) and (b) respectively (Table 3).

### Mutations in tyrosyl-tRNA synthetase cause Charcot-Marie-Tooth (CMT) disease

**Figure 4:**
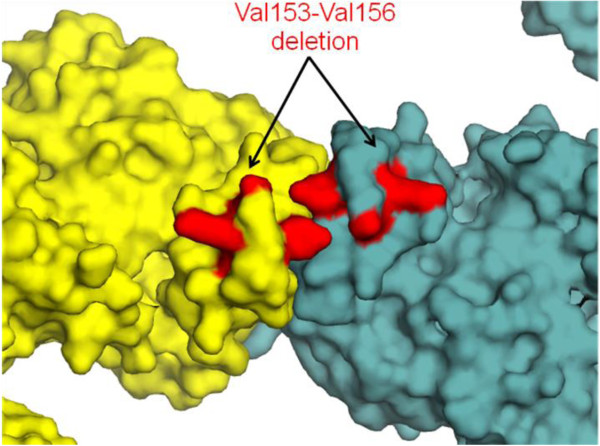
**Disease-associated deletion mutation (red) in YRS mapped onto the crystal structure with the two monomers shown in different colors.** Deletion mutation in human cytoplasmic YRS (red) mapped onto the corresponding regions in the crystal structure of *T. thermophilus* YRS (PDBID 1H3E).

Dysfunctional cytoplasmic YRS results because of two substitution and one deletion mutation (Table 1 and Figure 2B) resulting in Charcot-Marie-Tooth (CMT) disease [41]. Amongst heritable disorders of peripheral nervous system, CMT is the most prevalent disease [47, 48]. CMT can be of two types – type I is induced by axonal demyelination whereas type II results because of decreased amplitudes of evoked motor and sensory nerve responses [49]. Symptoms for CMT include muscular weakness, stoppage gait, high arched foot, reduced or absent deep-tendon reflexes, and impaired sensation [47, 49]. Two substitution mutations in YRS have been identified using genome-wide SNP analysis followed by PCR-RFLP (Polymerase Chain Reaction-Restriction Fragment Length Polymorphism) of selected candidate genes. We observed that the wild-type YRS residues at these sites are highly conserved in mammalian YRS sequences (Table 2). The Gly41Arg change occurs in a sector responsible for ATP recognition and hence potentially disrupts ATP binding to YRS (Figure 2B). This drastic mutation site occurs in a solvent accessible site where it forms part of β-strand structure within the catalytic domain (Figure 2B). In contrast, the Glu196Lys mutation site is buried as part of catalytic domain α-helix in protein core (Figure 2B and Table 2). Reversal of charge coupled with larger size of mutated residue (Glu196Lys) may alter the local physicochemical environment - thereby altering YRS structural stability. Finally, the four-residue deletion (Val153-Val156) occurs in a solvent exposed sector of α-helix H7 within the Rossmann fold domain and thereby likely affects the dimerization of YRS (Figure 4). Based on our annotation criteria, the Gly41Arg and Glu196Lys mutations belong to categories (a) and (b), respectively whereas the deletion mutation falls under category (c) (Table 3).

### Mutations in alanyl-tRNA synthetase that cause CMT and muscular neuropathy

Four ARS substitution mutations (Asn71Tyr, Arg329His, Glu778Ala and Asp893Asn) identified using exome sequencing are associated with CMT disease [42] (Table 1 and Figure 2B). Interestingly, for Glu778Ala and Asp893Asn the corresponding mutated residue is naturally present in other mammalian ARS sequences e.g. *B. taurus* ARS has Ala778 and *Mustela putorius furo* (domestic ferret) ARS has Asn893 (Table 2). Our results suggest that none of the four substitution mutations directly participate in inter-molecular interactions with ARS substrates (Figure 2B and Table 2). The buried site Asn71Tyr lies in catalytic domain β-strand structure where it may affect enzyme stability due to introduction of large hydrophobic residue in place of smaller hydrophilic one (Figure 2B). The Arg329His mutation occurs in a region of high sequence and structural conservation at a solvent inaccessible site within the ARS catalytic domain suggesting structural perturbation (Figure 2B). Previous biochemical studies have shown that ARSs with mutations of Asn71Tyr or Arg329His are defective in aminoacylation activity [42]. The Glu778Ala and Asp893Asn reside in C-terminal domain and there are no corresponding regions in the homologous crystal structure. Overall, the four ARS mutations were observed in different sub-domains of ARS and the two annotated mutations (Asn71Tyr and Arg329His) belong to category (b) whereas the other two mutations fall under category (d) (Table 3).

### Mutations in lysyl-tRNA synthetase cause CMT disease

**Figure 5:**
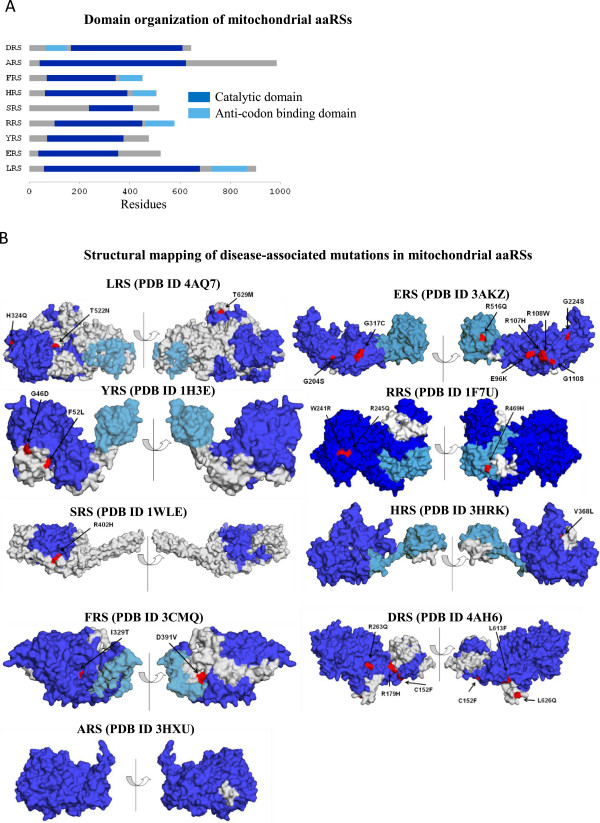
**Disease-associated mutations in human mitochondrial aaRSs. (A)** Schematic representation of domain architecture of mitochondrial aaRSs in which disease-associated substitution mutations have been identified. Catalytic domain and anticodon binding domain are in blue and cyan respectively. Domain boundaries for aaRS sequences were calculated using Pfam server. **(B)** Mutations (red) in cytoplasmic aaRSs mapped on the crystal structures where its domains have been color-coded corresponding to **(A)**.

Three KRS substitution mutations (Leu133His, Ile302Met, and Thr623Ser) identified using whole exome sequencing have been associated with CMT disease (Table 4 and Figure 5B) [45, 46]. *KARS* gene encodes two alternately spliced isoforms that catalyze aminoacylation in cytoplasm and mitochondria. Our sequence analyses suggest that the corresponding wild-type residues are fully conserved in mammalian KRSs (Table 5). We observe that the Leu133His mutation does not participate in any direct contacts with ATP, amino acid, tRNA or dimeric partner (Figure 5B). Interestingly, this mutation lies at a position with high sequence and structural conservation (>80%) and is part of a buried α-helix within the anti-codon binding domain suggesting that it might affect KRS structural stability (Table 5). Previous reports on functional aspects of the Leu133His mutation show that the mutant protein has severely compromised aminoacylation activity [45]. The Ile302Met adopts β-strand conformation within the catalytic domain and does not participate in inter-molecular interactions with any of the components of aminoacylation reaction (Figure 5B). It is likely that the introduction of this mutation causes steric incompatibility within the protein structure. For the third mutation (Thr623Ser) that lies towards C-terminal there is no corresponding residue in structural homologues. Overall, KRS mutations appear to affect structural stability of the enzyme leading to CMT and therefore these mutations fall in category (b) and (d). Finally, a frame shift mutation (Tyr173SerfsX7) within the anti-codon binding domain results in premature termination of the transcript, again linked to CMT [45].

**Table 4:** **List of disease-associated mutations in human mitochondrial aaRSs**

aaRS (class)	ID (state)	Substitution mutation(s)	Disease(s)/affected organ
LRS (I)	Q15031 (Monomer)	His324Gln, Thr522Asn, Thr629Met	Type-2 Diabetes, premature ovarian failure and hearing loss in Perrault syndrome/ovary and ears
ERS (I)	Q5JPH6 (Monomer)	Arg168Gly, Gly110Ser, Gly204Ser, Glu96Lys, Lys167Tyr, Gly317Cys, Arg55His, Gly224Ser, Arg107His, Arg108Trp, Arg516Gln	Leukoencephalopathy with thalamus and brainstem involvement and high lactate ‘LTBL’/brain
YRS (I)	Q9Y2Z4 (Dimer)	Gly46Asp, Phe52Leu	Myopathy, lactic acidosis, and sideroblastic anemia/muscles and blood cells
RRS (I)	Q5T160 (Monomer)	Ile9Val, Gln12Arg, Trp241Arg Arg245Gln, Arg469His	Ponto cerebellar hypoplasia type 6 (PCH6)/brain
SRS (II)	Q9NP81 (Dimer)	Asp390Gly, Arg402His	HUPRA syndrome/lungs and kidneys
HRS (II)	P49590 (Dimer)	Val368Leu, Leu200Val	Perrault syndrome, ovarian dysgenesis and sensorineural hearing loss or Perrault syndrome/ovary and ears
FRS (II)	O95363 (Dimer)	Ile329Thr, Asp391Val, Tyr144Cys	Fatal infantile alpers encephalopathy, mitochondrial myopathies, diabetes, encephalopathies, and deafness/central nervous system disease, muscles and brain
ARS (II)	Q5JTZ9 (Monomer)	Leu155Arg and Arg592Trp	Infantile cardiomyopathies/heart in new born babies
DRS (II)	Q6PI48 (Dimer)	Ser45Gly, Cys152Phe, Arg179His, Leu239Pro, Arg263Gln, Leu613Phe, Leu626Gln, Leu626Val	Leukoencephalopathy with brainstem and spinal cord involvement and elevated lactate/brain

**Table 5:** **Annotations for mitochondrial aaRSs**

aaRS	Mutation	Sequence spread in homologs (%)	Structure	RSA	Intermolecular interaction	Domain	Reference
LRS (I)	His324Gln	‘H’: 48, ‘K’: 22, ‘C’: 14, ‘-’: 4, ‘R’: 4, ‘N’: 3, ‘P’: 3, ‘A’: 1, ‘G’: 1	H	**4.7**	-	Catalytic	[50, 51]
	Thr522Asn	‘T’: 86, ‘-’: 14	T	20.4	-	Catalytic	
	Thr629Met	‘T’: 63, ‘L’: 22, ‘-’: 14, ‘V’: 1	E	**11.6**	-	Catalytic	
ERS (I)	Arg55His	‘R’: 77, ‘K’: 12, ‘-’: 10, ‘L’: 1	H	**4.4**	-	Catalytic	[52]
	Glu96Lys	‘E’: 79, ‘A’: 10, ‘-’: 8, ‘T’: 3	H	64.9	-	Catalytic	
	Arg107His	**‘R’: 79, ‘-’: 15, ‘H’: 2, ‘T’: 2, ‘C’: 1, ‘Q’: 1**	T	47.8	-	Catalytic	
	Arg108Trp	‘R’: 78, ‘-’: 15, ‘Q’: 4, ‘E’: 3	T	56.8	-	Catalytic	
	Gly110Ser	‘G’: 84, ‘-’: 15, ‘E’: 1	-	52.8	-	Catalytic	
	Cys167Tyr	‘C’: 83, ‘-’: 15, ‘Q’: 1, ‘R’: 1	-	-	-	Catalytic	
	Arg168Gly	‘R’: 83, ‘-’: 14, ‘L’: 2, ‘K’: 1	-	-	-	Catalytic	
	Gly204Ser	‘G’: 82, ‘Y’: 11, ‘-’: 6, ‘V’: 1	E	38.1	-	Catalytic	
	Gly224Ser	‘G’: 81, ‘-’: 17, ‘A’: 1, ‘V’: 1	S	42.8	-	Catalytic	
	Gly317Cys	‘G’: 81, ‘T’: 10, ‘-’: 7, ‘S’: 2	-	44.7	tRNA binding	Catalytic	
	Arg516Gln	‘R’: 71, ‘-’: 11, ‘D’: 10, ‘S’: 3, ‘E’: 2, ‘K’: 2, ‘V’: 1	H	**6.4**	-	C-terminal	
YRS (I)	Gly46Asp	‘G’: 84, ‘-’: 12, ‘Q’: 2, ‘E’: 1, ‘S’: 1	T	52.3	-	-	[53, 54]
	Phe52Leu	‘F’: 89, ‘-’: 10, ‘Y’: 1	E	28.8	-	-	
RRS (I)	Ile9Val	‘I’: 84, ‘-’: 12, ‘A’: 1, ‘E’: 1, ‘F’: 1, ‘L’: 1	-	-	-	-	[55]
	Gln12Arg	**‘Q’: 53, ‘E’: 25, ‘-’: 11, ‘K’: 6, ‘T’: 3, ‘L’: 1, ‘R‘: 1**	-	-	-	-	
	Trp421Arg	‘W’: 98, ‘-’: 2	H	**13.2**	-	Catalytic	
	Arg245Gln	‘R’: 98, ‘-’: 2	H	**6.8**	-	Catalytic	
	Arg469His	‘R’: 99, ‘-’: 1	H	23.4	tRNA binding	Editing	
SRS (II)	Asp390Gly	‘D’: 78, ‘N’: 14, ‘-’: 7, ‘E’: 1	E	**1.2**	-	Catalytic	[56]
	Arg402His	‘R’: 78, ‘K’: 14, ‘-’: 7, ‘A’: 1	E	**14.9**	-	Catalytic	
HRS (II)	Leu200Val	‘L’: 100	H	**0.0**	-	Catalytic	[57]
	Val368Leu	‘V’: 100	H	**1.2**	-	Catalytic	
FRS (II)	Tyr144Cys	‘Y’: 73, ‘-’: 19, ‘F’: 6, ‘E’: 1, ‘L’: 1	-	**0.9**	-	Catalytic	[58]
	Ile329Thr	‘I’: 73, ‘-’: 16, ‘W’: 6, ‘L’: 3, ‘G’: 1, ‘V’: 1	G	**4.1**	-	Catalytic	
	Asp391Val	‘D’: 81, ‘-’: 16, ‘G’: 2, ‘E’: 1	G	26.3	-	-	
ARS (II)	Leu155Arg	‘L’: 97, ‘-’: 3	H	**0.0**	-	Catalytic	[59]
	Arg592Trp	‘R’: 51, ‘Q’: 20, ‘N’: 20, ‘G’: 6, ‘-’: 3	-	-	-	Catalytic	
DRS (II)	Ser45Gly	‘S’: 76, ‘-’: 14, ‘N’: 8, ‘E’: 1, ‘K’: 1	S	**10.7**	Dimerization	-	[27, 60–63]
	Cys152Phe	‘C’: 74, ‘A’: 16, ‘-’: 6, ‘Q’: 1, ‘S’: 1, ‘W’: 1, ‘X’: 1	-	31.1	Dimerization	-	
	Arg179His	‘R’: 92, ‘-’: 7, ‘G’: 1	T	28.6	-	Catalytic	
	Leu239Pro	‘L’: 93, ‘-’: 7	-	**18.2**	-	Catalytic	
	Arg263Gln	‘R’: 79, ‘P’: 16, ‘-’: 5	E	56.0	Dimerization	Catalytic	
	Leu613Phe	‘L’: 74, ‘P’: 15, ‘-’: 11	S	41.4	tRNA binding	-	
	Leu626Gln	‘L’: 73, ‘-’: 25, ‘I’: 2	T	64.0	-	-	
	Leu626Val	‘L’: 73, ‘-’: 25, ‘I’: 2	T	64.0	-	-	

Mutations that were observed to be naturally present in corresponding aaRSs are highlighted (**bold**). RSA values (%) in bold indicates buried positions. SS and RSA could not be assigned for residues that were disordered in the crystal structure. Also see Figure 5.

#### Mutational landscape of mitochondrial aaRSs

Mitochondria are ATP synthesizing organelles in eukaryotic cells. The 16,569 base pairs long closed circular human mitochondrial DNA encodes for proteins, rRNA and tRNA [64]. Additional nuclear encoded proteins required for protein synthesis such as aaRSs and transcription factors are imported into mitochondria [64]. Dysfunctional mitochondria, due to mutations in mitochondrial or nuclear DNA (including in tRNA genes and those that encode mitochondrial proteins), have been implicated in numerous human diseases [28, 65–67]. Remarkably, the pathologies of dysfunctional mitochondria aaRSs are not restricted to systematic impairment of ATP synthesis but rather seem to have tissue-specific phenotypes [23]. Mutations have been identified in both Class I and II enzymes. Most human diseases attributable to mutations in aaRSs are due to alterations in the mitochondrial copies of aaRSs. We show that ~55% of mitochondrial aaRSs have been implicated in disease-associated mutations compared with ~20% for cytoplasmic aaRSs (Table 4 and Figure 1B). Each point mutation in a given mitochondrial aaRS has been independently associated with a given disease except in cases of RRS and HRS where more than one substitution has been observed in a single patient (see Table 5). Figure 5A shows schematic representations of mitochondrial aaRS domain architectures. Specific analyses of mutations follow here:

### Mutations in mito-leucyl-tRNA synthetase causes ovarian failure and diabetes

Three substitution mutations (His324Gln, Thr522Asn and Thr629Met) in the mitochondrial LRS have been associated with ovarian failure, hearing loss and type-2 diabetes (Table 4 and Figure 5B). Multiple sequence alignment of homologous LRS sequences reveals that the wild-type residues for these are fully conserved across mammalian LRSs (Table 5). We observed that none of these mutations are likely to directly interact with aminoacylation reaction substrates (Figure 5B and Table 5). The position for His324Gln mutation lies buried as part of α-helical region (H12 in PDB ID 4AQ7) in the catalytic domain and shows ~60% structural conservation within class I aaRSs. Thr522Asn and Thr629Met are located in buried sites within the LRS catalytic domain and adopt turn and β-strand conformations respectively. In summary, our structural analysis suggests that all the three LRS mutation falls into category (b).

### Mutations in mito-tyrosyl-tRNA synthetase cause MLASA

Two substitution mutations (Gly46Asp and Phe52Leu) in the YRS Rossmann fold domain have been linked to myopathy, lactic acidosis and sideroblastic anemia (MLASA) [53, 54]. We observed that in each of these cases, the corresponding wild-type residues are fully conserved in YRSs (Table 5). Further investigation into location of mutations suggests a lack of direct interactions with YRS substrates or participation in enzyme dimerization (Figure 5B). The Gly46Asp and Phe52Leu mutations are solvent accessible and occur in β-strand and turn regions respectively within the N-terminal region (and distal from the catalytic sector). It therefore remains unclear from our analyses how these mutations in non-functional YRS regions lead to disease states in humans (category d). Previous biochemical studies have shown that the mutant YRSs have abnormal kinetics *vis-a-vis* wild-type enzyme [53].

### Mutations in mito-alayl-tRNA synthetase cause cardiomyopathy

Two substitution mutations (Leu155Arg and Arg592Trp) in ARS identified using whole exome sequencing are associated with cardiomyopathies in infants [59] (Table 4 and Figure 5B). Comparison of the mammalian ARS sequences suggests very high conservation of these two ARS sites. The Leu155Arg change is part of α-helix (H4 in Rossmann fold domain), remains buried and given distance considerations it is unlikely to affect inter-molecular substrate contacts (Figure 5B). The drastic alteration in physicochemical environment (replacement of leucine with positively charged arginine) may affect ARS folding and stability. The other ARS mutation (Arg592Trp) occurs within the editing domain and is severe given the conversion of arginine into a large aromatic residue. Hence, both the mutations are likely to reduce structural stability of ARS and therefore fall in category (b).

### Mutations in mito-glutamyl-tRNA synthetase cause leukoencephalopathy

**Figure 6:**
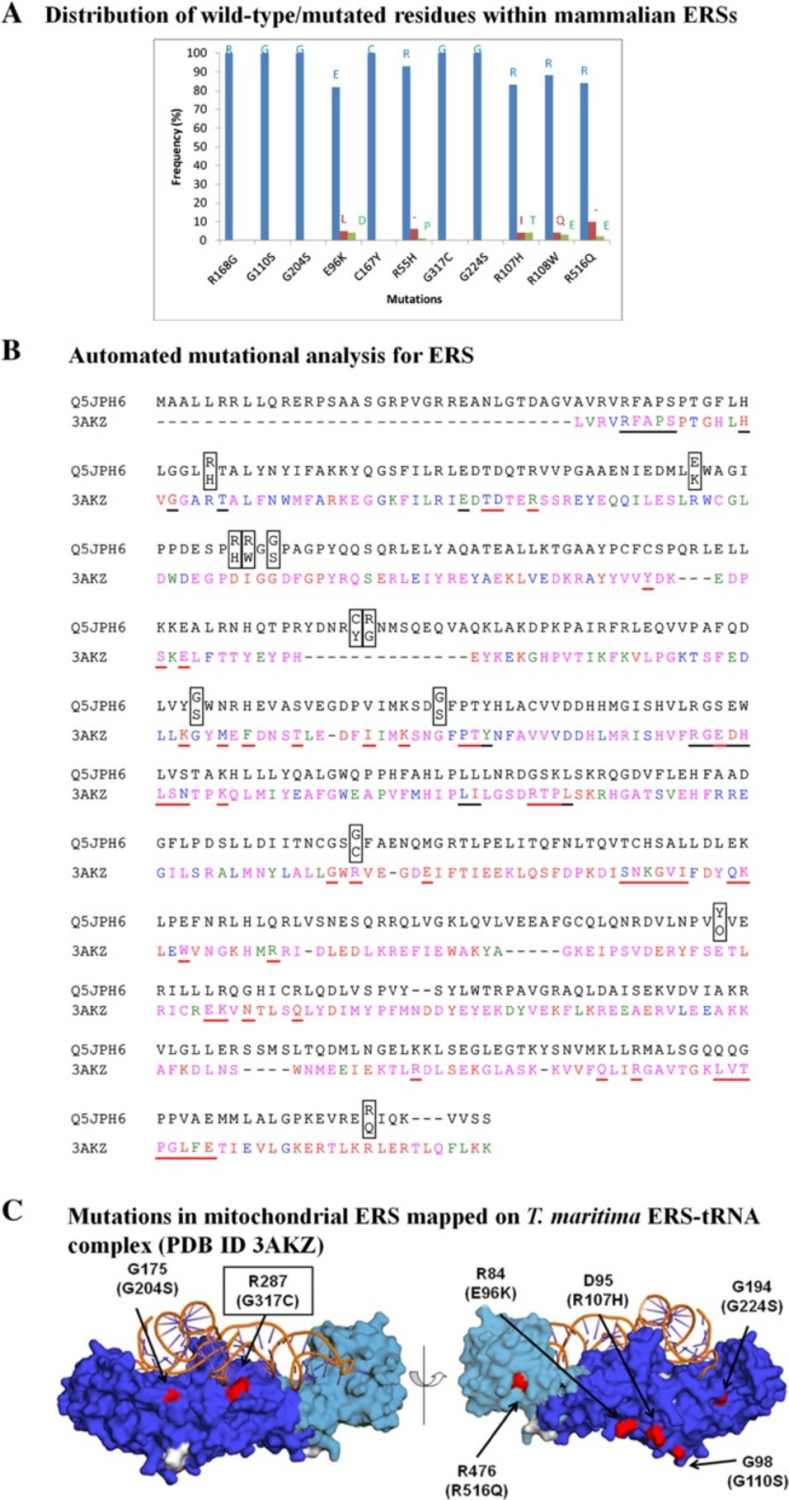
**Annotation for mutations in human mitochondrial ERS. (A)** Frequency distribution of residues at sites of ERS mutations. The distribution was calculated based on top 100 mammalian homologous sequences identified using BLAST. A ‘-’ represents frequency for gaps in the alignment. **(B)** Sequence alignment of mitochondrial ERS (UniProt ID Q5JPH6) and *T. maritima* ERS where substitutions in human protein are shown in boxes. Residues in *T. maritima* ERS that interact with ATP/analogue and tRNA are underlined in black and red respectively. Structural conservation for *T. maritima* ERS is graded in four categories <40%, 40-60%, 60-80%, and >80% which are highlighted as magenta, blue, green and red respectively. **(C)** Mutations (in parentheses) in human ERS mapped on to the crystal structure of *T. maritima* ERS where mutation in tRNA-binding interface is highlighted with boxed labels.

ERS mutations are associated with multiple pathologies including myopathy, respiratory failure and retinitis pigmentosa [52]. A total of 14 substitution mutations and one insertion mutation (Thr426_Arg427insL) have been reported for ERS (Table 4). Only one of the substitution mutations, Arg107His, is naturally present in mitochondrial ERS from *Myotis davidii* (mouse-eared bat) (Table 5 and Figure 6A). All the other mutations map to evolutionarily conserved positions within the ERS family (Figure 6B). Four substitution mutations (Arg107His, Arg108Trp, Gly110Ser, and Arg516Gln) are observed at positions with very high structural conservation (>80%, residues are red in Figure 6B) and therefore may affect enzyme structural core. In addition, the loss of positive charge at three out of these four positions would alter the electrostatic properties of ERS. The Gly317Cys mutation occurs in the tRNA binding sector and could potentially affect tRNA binding to ERS (Figure 6C). Four additional mutations – Arg55His, Glu96Lys (catalytic domain), Arg516Gln (C-terminal domain), and a deletion mutation at position 398 (catalytic domain) occur in α-helical structures of ERS. Amongst these substitution mutations, Arg55His and Arg516Gln are buried and therefore could potentially alter enzyme stability. In cases of the Arg107His and Arg108Trp mutations, the substitution of exposed arginines will likely alter ERS electrostatic properties. Taken together, the ERS mutations fall within two different categories – (a) and (b) (Table 3).

### Mutations in mito-arginyl-tRNA synthetase cause Ponto cerebellar hypoplasia

Five substitution mutations and one frame shift mutation have been identified in human RRS (Table 4 and Figure 5B) [55]. Our annotation analysis suggests that none of the five substitutions are likely to directly contact substrate atoms (Figure 5B). Of the five, sequence comparisons show that Gln12Arg mutation is naturally present in rabbit RRS sequence (Table 5) suggesting that this mutation may not alter enzyme activity as it is tolerated in other mammals. However, in case of other three - Trp241Arg, Arg245Gln and Arg469His the wild-type residues are highly conserved and these form part of an α-helix. The Trp241Arg and Arg245Gln substitutions occur at buried sites that show high structural conservation (>80%) within the Rossmann fold domain – hence these are likely to cause structural perturbation of RRS (category b) (Figure 5B). Further, the Arg469His lies in the tRNA binding region of RRS editing domain and hence falls in category (a). For the remaining two N-terminal mutations (Ile9Val and Gln12Arg) our analyses suggest structural disorder in homologs and hence these remain un-annotated (category d). Amongst the five disease-associated mutations in RRS, Arg245Gln and Arg469His substitutions were observed in single patient family; similarly, Gln12Arg and Trp241Arg substitutions were identified within one disease affected family [55].

### Mutations in mito-seryl-tRNA synthetase cause several disorders

The Asp390Gly and Arg402His mutations in SRS are associated with fatalities in newborns [56] (Table 4 and Figure 5B). Patients with the HUPRA (Hyperuricemia, pulmonary hypertension, renal failure, and alkalosis) syndrome generally die because of multi-organ failure, respiratory insufficiency or lung hypertension – these SRS mutations were identified using SNP microarray analysis [56]. Our analysis suggests that mutation hot sites occur in residues of high conservation across mammalian SRSs (Table 5). The Asp390Gly and Arg302His changes occur in buried sites within catalytic domain of SRS, although they likely do not contact enzyme substrates (Figure 5B). Interestingly, it has been reported that the Asp390Gly mutant protein exhibits lower level of aminoacylation activity for one of the (two) isoacceptor tRNA^Ser^[56]. However, it seems that the Asp390Gly mutation does not affect charging of the other isoacceptor tRNA^Ser^_UCN_[56]. These data present an enigma as they suggest differential recognition of cognate tRNAs by the Asp390Gly mutant protein. Nonetheless, the Asp390Gly mutation leads to loss of function leading the pathological condition. From our analysis, it is evident that both these mutations are in category (b).

### Mutations in mito-histidyl-tRNA synthetase cause Perrault syndrome

**Figure 7:**
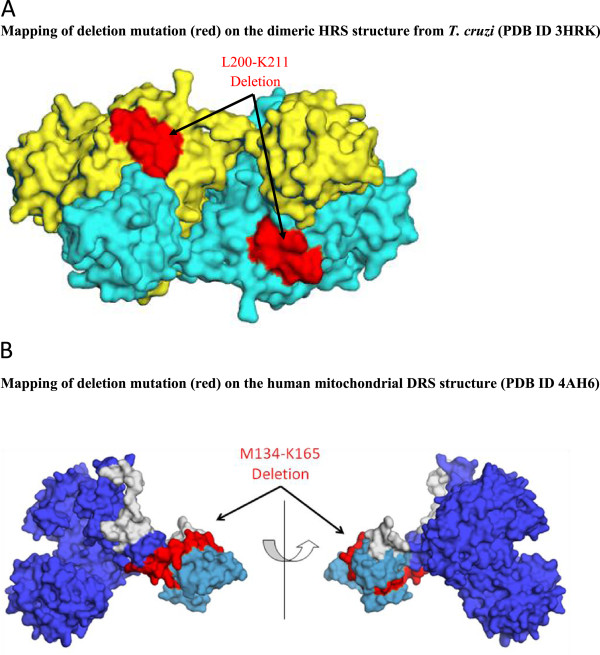
**Disease-associated deletion mutations (red) in mitochondrial HRS (A) and DRS (B) mapped onto their corresponding crystal structures.**

HRS mutations identified using genomic sequencing are associated with ovarian dysgenesis and Perrault syndromes [57]. Two substitution mutations (Leu200Val and Val368Leu) and one deletion mutation in HRS have been implicated (Table 4 and Figure 5B). The affected individual was observed to harbor both these mutations in HRS simultaneously. Sequence analyses within mammalian HRSs reveal high conservation of wild type residues at these positions (Table 5), suggesting evolutionarily important functional roles. Both Leu200Val and Val368Leu form part of α-helix, where the former locates in HRS catalytic domain while the latter is at the junction of Rossmann fold domain and the loop connecting C-terminal domain (Figure 5B). Our analysis shows that both these mutation sites are buried, and hence it is unlikely that these participate in direct contacts with either the enzyme substrates or its dimeric partner. These observations suggest that both these mutations fall under category (b). Finally, a deletion mutant where residues Leu200 to Lys211 are missing is associated with Perrault syndrome [57]. These residues (200 to 211) constitute a turn and a β-strand structure within the dimeric interface of HRS (Figure 7A). It is likely that the deletion mutant protein has severely compromised ability to form dimers (category c) and hence impaired enzymatic activity.

### Mutations in mito-phenylalanyl-tRNA synthetase cause muscular and neurological disorders

Three FRS substitution mutations identified using whole exome sequencing have been implicated in various neurological and metabolic diseases in humans [58] (Table 4 and Figure 5B). Wild-type positions at mutant sites display very high sequence conservation (>90%) within mammalian FRSs (Table 5). Our structural evaluations suggest that the Ile329Thr mutation within the catalytic domain is likely to make direct contacts with ATP (Figure 5B). The Ile329Thr and Asp391Val (in C-terminal domain) sites are present in 3_10_ helical regions of FRS (Figure 5B). The third substitution mutation (Tyr144Cys) is buried and is not part of a well-defined secondary structure element within the protein. Interestingly, the site for Tyr144Cys mutation suggests high structural conservation (>80%). Overall, our results are consistent with previous reports that correlate these FRS mutations to disease states by affecting aminoacylation activity as well as destabilizing the structure of FRS [58]. Hence, the three FRS mutations fall within categories (a) and (b).

### Mutations in mito-aspartyl-tRNA synthetase cause leukoencephalopathy

Autosomal recessive mutations in mitochondrial DRS induce LBSL (Leukoencephalopathy with brainstem and spinal cord involvement and elevated lactate) [68, 69] that is characterized by abnormal muscle stiffness (spasticity) and difficulty with coordinating movements (ataxia) [68, 69]. Ten substitution mutations, one frame shift mutation, and one deletion mutation have been identified in DRS (Table 4 and Figure 5B). These sites are highly conserved across mammalian DRSs (Table 5). Four substitution mutations - Ser45Gly, Cys152Phe, Arg179His, and Arg263Gln are at the dimeric interface of DRS (Figure 5B). It is likely that these mutations disrupt the network of non-bonded interactions at the dimeric interface leading to dysfunctional DRS. The Leu613Phe mutation occurs in the tRNA binding region - however this mutation has been shown to have no affect on enzyme activity [70]. The site for Leu626Gln mutation has very high structural conservation (>80%) indicating that substitution at this site might perturb DRS structure. Two nonsense mutations at positions Arg263 and Glu425 result in the introduction of a stop codon leading to inactive protein. One deletion within the anticodon-binding domain ranging from Met134 to Lys165 has also been associated with LBSL in humans (Figure 7B) [69]. Overall, our mutational annotations suggest that the pathological consequences of dysfunctional DRS could stem from various molecular abnormalities including defective dimerization, incompetent tRNA binding and structural instability. The DRS mutations hence fall under categories (a), (b), and (c) (Table 3).

## Conclusions

We have developed a mutational annotation pipeline that functionally categorizes mutations in proteins. The pairwise sequence alignment, which is the primary output from our analysis pipeline, pictorially provides valuable structural and evolutionary sequence conservation information. Our approach also offers rapid structural annotation for mutations in proteins vis-à-vis methods that first build a 3D model of a sequence followed by mapping of the mutations. Our methodology can be automated to annotate and classify mutations in any protein family. In addition to the annotations adopted in this study, customized annotations can also be included in the output e.g., the pairwise sequence alignment can have annotations for residues interacting with biomolecular partner(s). The approach presented here is built on an open-source platform that is freely available for academic research, and it provides a facile tool to decipher molecular effects of mutations in proteins.

Mutations in proteins can cause pathological effects through a variety of molecular mechanisms. Integrated computational and structural biology offers an opportunity to analyze molecular phenotypes for disease-associated mutations in a high-throughput manner within structural contexts of mutant proteins. The aaRSs are ubiquitous enzymes that drive protein translation in cells [5]. These enzymes accurately catalyze charging of tRNAs with their cognate amino acids and therefore effectively decode the genetic code [5, 7]. Mutations in aaRSs lead to a variety of diseases in humans [28]. Considering the diverse roles of aaRSs, it is likely that mutations within these proteins could manifest their pathological phenotypes by either affecting the canonical activities (aminoacylation and/or editing) or non-canonical functions (gain- or loss-of-function). The etiology of these mutant phenotypes could involve multitude of facets such as altered inter-molecular interactions; abnormal sub-cellular localization; changes in oligomeric states; variations in structural stability or protein aggregation tendencies.

**Figure 8:**
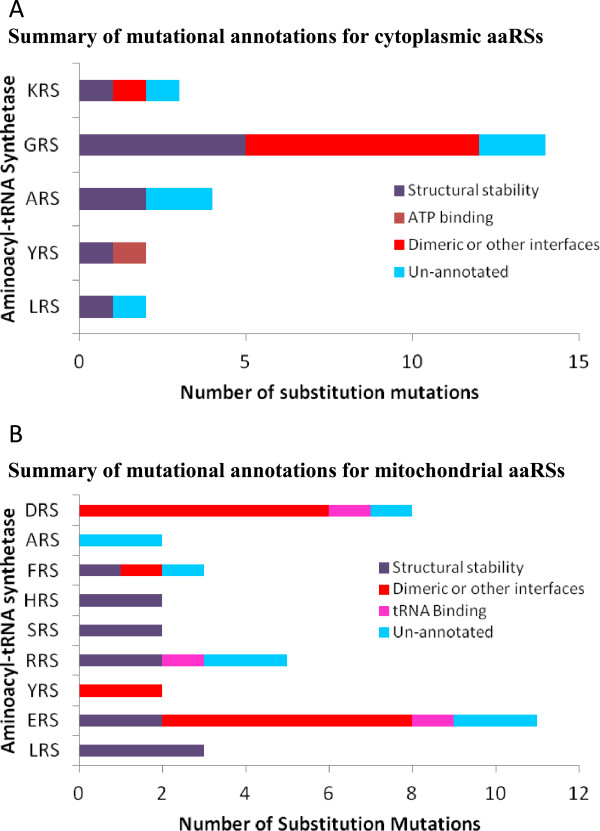
**Summary for annotation for mutations in aaRSs within cytoplasm (A) and mitochondria (B) using our mutational annotation pipeline.**

In this study, we have performed systematic and rigorous computational analyses of human aaRSs disease-associated mutations to evaluate their evolutionary, structural and functional characteristics. Our results show that aaRS mutations can occur in structurally conserved regions within both cytoplasmic and mitochondrial aaRSs. Four mutations in cytoplasmic and seven in mitochondrial aaRSs were observed to participate in inter-molecular interactions with substrates of aminoacylation reactions such as ATP, amino acid or tRNA (Figure 6). Such mutations therefore could directly manipulate the ability of aaRSs to accurately execute enzymatic activities potentially leading to slower kinetics or even loss of activity. In addition, mutational alterations distal from the active site regions could affect non-canonical functions that are often associated with this protein family (Table 3). Evidence supporting pathological effects of mutations due to anomalous non-canonical aaRS functions comes from mouse model for CMT where a mutant GRS retains wild-type aminoacylation activity [71]. Apart from mutations annotation, we also show that deletion mutations in YRS and HRS likely disrupt their dimeric assemblies, which in turn would lead to stalling of new protein translation (Figures 4 and 8A). Similarly, the deletion mutations in ERS and DRS could compromise ERS-tRNA interactions and editing activity respectively (Figures 6C and 8B).

In humans, 9 aaRSs and 3 auxiliary proteins assemble to form multisynthetase complex (MSC) within the cytoplasm [72]. These MSCs plays are critical role in various non-translational activities within the cell. Cytoplasmic LRS is the only component of MSC for which disease-associated mutations have been reported [40]. It has been suggested that the C-terminal domain of LRS interacts with RRS within MSC [73]. Our analysis shows that disease-associated mutations in LRS localize within the N-terminal catalytic domain, and thus may not have a direct affect on the assembly of MSC. Further crystallographic studies on MSC would provide necessary insights into the assembly of different synthetases within these complexes, as has been achieved for other cellular assemblies [74–76].

Out of 63 mutations annotated in this work, only 12 (~20%) were observed in regions that could directly affect aminoacylation activity via either binding to ATP/ amino-acid, tRNA or by involvement in dimerization. Mutations in structural cores or at potential biomolecular interfaces account for 55% mutations while remaining mutations (25%) remain structurally un-annotated (Figure 8). These observations call for further experimental investigations to understand the molecular effects caused by mutations in aaRSs. Overall, the landscape for mutated aaRSs highlights that no particular site within aaRSs is specifically prone to mutations, or seems mutated often. Amongst cytoplasmic and mitochondrial aaRSs, the most frequently mutated residues were glycine (smallest residue, 6 mutations) and arginine (largest polar residue, 10 mutations) respectively. Further, Gly to Arg, Arg to His, and Arg to Gln were the three most frequent substitution mutations in aaRSs (Tables 1 and 4). Intriguingly, only ~20% of cytoplasmic aaRSs have been reported to harbor disease-associated mutations compared to ~55% in mitochondrial aaRSs (Figure 1A and 1B). Understanding the determinants for higher propensity of mutations in mitochondrial aaRSs within the nuclear genome requires additional functional and genetic data. Such thrusts would advance our understanding of heritable disorders since mitochondria plays a critical role in maternal inheritance and has been implicated in numerous human diseases.

## Electronic supplementary material

Additional file 1: Figure S1: Outline for the mutational annotation pipeline. (PDF 194 KB)
