# Supplementary figures and images for: Evolutionary and structural annotation of disease-associated mutations in human aminoacyl-tRNA synthetases
Source: BMC Genomics. 2014 Dec 4;15(1):1063. doi: 10.1186/1471-2164-15-1063 (PMC4298046; doi:10.1186/1471-2164-15-1063)

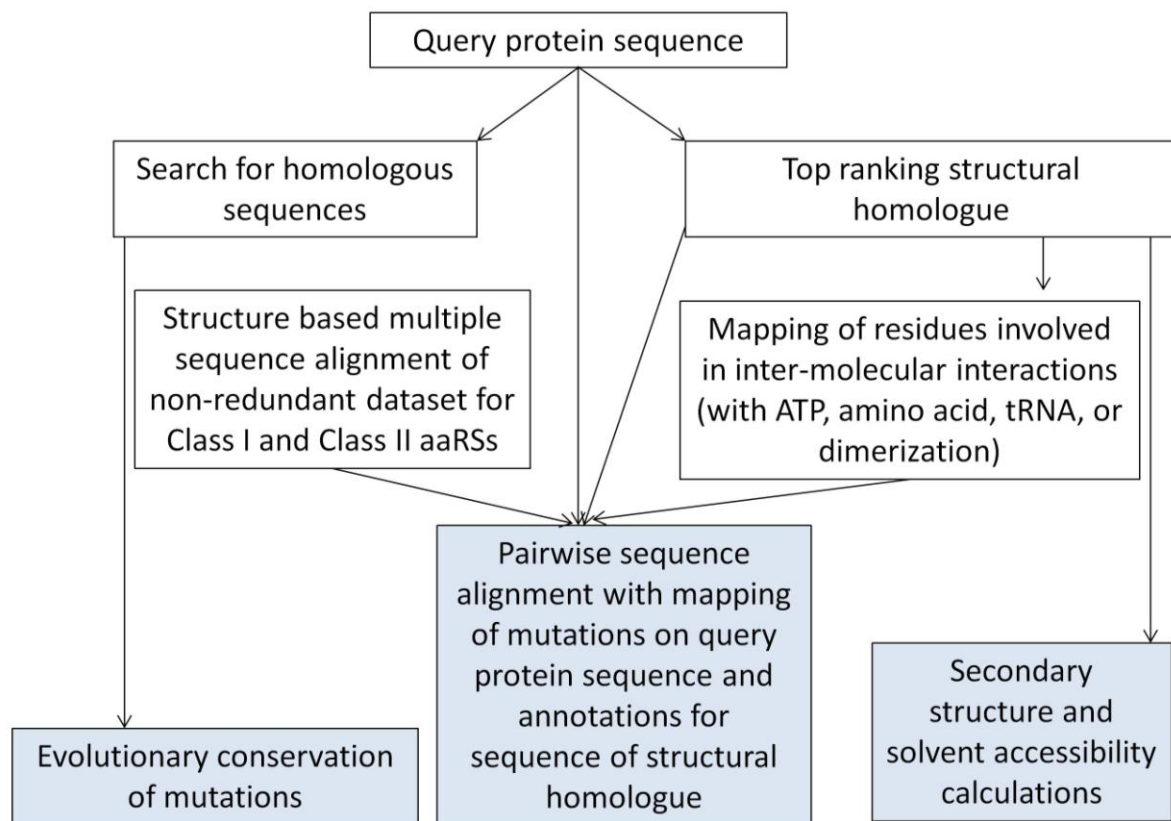

Figure S1: Outline for the mutational annotation pipeline.

Supplement: Supplementary file 1 — Additional file 1: Figure S1: Outline for the mutational annotation pipeline. (PDF 194 KB) [file 12864_2014_6929_MOESM1_ESM.pdf]
